# PGC1β Regulates Breast Tumor Growth and Metastasis by SREBP1-Mediated HKDC1 Expression

**DOI:** 10.3389/fonc.2019.00290

**Published:** 2019-04-17

**Authors:** Xiaoli Chen, Yang Lv, Ying Sun, Hongyu Zhang, Weiguo Xie, Liyan Zhong, Qi Chen, Min Li, Ling Li, Jia Feng, Athena Yao, Qi Zhang, Xiaodong Huang, Zhendong Yu, Paul Yao

**Affiliations:** ^1^Institute of Rehabilitation Center, Tongren Hospital of Wuhan University (Wuhan Third Hospital), Wuhan, China; ^2^Hainan Maternal and Child Health Hospital, Haikou, China; ^3^Peking University Shenzhen Hospital, Shenzhen, China; ^4^Department of Neurology, Renmin Hospital of Wuhan University, Wuhan, China

**Keywords:** hexokinase, HKDC1, mitochondria, PGC1β, SREBP1

## Abstract

**Background:** Breast cancer is a very common cancer with significant premature mortality in women. In this study, we show that HKDC1 expression in breast cancer cells is increased significantly. We aim to investigate the detailed mechanism for the regulation of HKDC1 expression and its potential contribution to tumorigenesis.

**Methods:** Gene expression was evaluated by real time PCR, western blotting, and immunohistochemistry. The mechanism for PGC1β/SREBP1-mediated HKDC1 expression was investigated using luciferase reporter assay, chromatin immunoprecipitation, and siRNA techniques. In addition, HKDC1 was overexpressed or knocked down by lentivirus to evaluate the potential effect on *in vitro* cell proliferation, glucose uptake, mitochondrial function, apoptosis, and reactive oxygen species (ROS) formation. Furthermore, an *in vivo* xenograft tumor development study was employed to investigate the effect of HKDC1 on tumor growth and mouse survival.

**Results:** HKDC1 is highly expressed in both breast cancer cells and clinical tumor tissues. HKDC1 expression is upregulated and co-activated by PGC1β through SREBP1 binding motif on the HKDC1 promoter. HKDC1 is located on the mitochondrial membrane and regulates the permeability transition pore opening by binding with VDAC1, subsequently modulating glucose uptake and cell proliferation. Overexpression of HKDC1 increases while knockdown of HKDC1 decreases *in vitro* breast cancer cell proliferation and *in vivo* tumor growth, metastasis, and mouse survival.

**Conclusions:** PGC1β regulates breast cancer tumor growth and metastasis by SREBP1-mediated HKDC1 expression. This provides a novel therapeutic strategy through targeting the PGC1β/HKDC1 signaling pathway for breast cancer treatment.

## Introduction

Breast cancer is a very common cancer with significant premature mortality in women. Around 12% of women in USA will have chance to be diagnosed with breast cancer during their lifetimes (1, 2). The development of breast cancer is regulated by many factors, and even as average survival rates have increased significantly as a result of many advanced treatments, the exact detailed mechanism of breast cancer development is still largely unknown (3).

The peroxisome proliferator-activated receptor-γ (PPARγ) co-activator-1β (PGC1β) is involved with tumor growth and metastasis, promoting tumorigenesis by modulation of mitochondrial function and glycolysis metabolism (4–8). PGC1β knockdown impairs ERRα signaling and reduces cell proliferation, suggesting a potential role of PGC1β in the pathogenesis of breast cancers (6). Sterol regulatory element-binding proteins (SREBPs) regulate many genes involved in lipid metabolism (9), and this process requires the expression of PGC1β (10). Three isoforms of SREBPs, including SREBP-1a,−1c, and−2, have been described, where SREBP-1a and SREBP-1c are encoded by a single gene with alternative splicing and promoters (11), while SREBP-2 is encoded by a different gene (12). SREBPs are key regulators of nutritional homeostasis (13, 14).

Hexokinase (HK) is the rate-limiting enzyme that converts glucose into glucose-6-phosphate, thus regulates the glucose metabolism in a wide variety of organisms (15). Four different kinds of hexokinase enzymes, including HK1, HK2, HK3, and HK4 [also known as glucokinase (GCK)] (16, 17), have been described with extensive characterization. Hexokinase plays a critical role in glycolysis; furthermore, increasing evidence demonstrates that that hexokinase is involved with cell proliferation and tumor growth (18). Aberrant expression of HK enzymes have been identified in many tumors (19). Hexokinase domain component 1 (HKDC1) has been recently discovered as a putative fifth hexokinase. The expression pattern of HKDC1 in the human body has been studied extensively as it regulates glucose homeostasis (15, 20, 21). Recent studies show that HKDC1 may be a novel potential therapeutic target for cancer (18, 22–24), although the detailed mechanism behind the role of HKDC1 in this process remains unclear.

Our preliminary data showed that HKDC1 expression is significantly increased in breast cancer cells and clinical tissues, while the mechanism remains unknown. In this study, we aim to investigate the potential effect and mechanism of HKDC1 on the contribution of breast cancer. We showed that HKDC1 is co-activated by PGC1β through a SREBP1 (25) binding motif located at−1286 (starting from transcription start site) on the HKDC1 promoter. We further investigated the gene expression of those co-activators, found that SREBP1 expression has no change, but PGC1β expression is significantly increased in breast cancer. HKDC1 overexpression significantly promotes cell proliferation in breast cancer cells, whereas HKDC1 knockdown suppressed this effect. In addition, we showed that HKDC1 is located in the mitochondrial outer membrane; it binds with VDAC1 (voltage dependent anion channel 1) (26), and modulates the mitochondrial transition pore, subsequently modulating glucose uptake (27). Further *in vivo* tumor xenograft studies showed that HKDC1 overexpression promoted tumor colony formation and resulted in decreased mouse survival, while HKDC1 knockdown reversed this effect. It is the first time that the mechanism behind the role of HKDC1 in tumor development in breast cancer has been identified, leading to the possibility that HKDC1 could be a potential target for cancer therapy.

## Materials and Methods

An expanded Materials and Methods section is available in Data S1.

### Materials and Reagents

Human primary mammalian epithelial cells (HMECs, obtained from Lonza) were cultured in MEGM BulletKit (CC-3150). MCF7 and MDA-MB-231 (MDA231, obtained from ATCC) were cultured in DMEM at 37°C supplemented with 10% FBS and antibiotics. Antibodies for β-actin (sc-47778), Ki-67 (sc-101861), SREBP1 (sc-13551), SREBP2 (sc-13552), and VDAC1 (sc-390996) were obtained from Santa Cruz Biotechnology. Antibodies for HKDC1 (ab228729) and PGC1β (ab176328) were obtained from Abcam. 3-nitrotyrosine (3-NT) was measured using the 3-Nitrotyrosine ELISA Kit (ab116691 from Abcam). The Coomassie Protein Assay Kit (Pierce Biotechnology) was used to measure the protein concentration. The siRNA for SREBP1, SREBP2, PGC1β, and negative control (#AM4636) were purchased from Ambion. The Lipofectamine™ Reagent (Invitrogen) was used for DNA transfection (5).

### Construction of HKDC1 Reporter Plasmids

The HKDC1 promoter (2000 bp upstream of TSS + first exon) from the Ensembl Transcription ID: HKDC1-201 (ENST00000354624.5) was amplified from human genomic DNA in HMEC cells by PCR using the following primers with the introduction of *Kpn* I/*Hind III* restriction sites as indicated by underline, HKDC1 Forward: 5′-gcgc-GGTACC-gaa aag gat ggg gat cct caa-3′ (*Kpn* I) and HKDC1 Reverse: 5′-gcgc-AAGCTT-ctt ctt gat ctg gtc ctc ctt-3′(*Hind III*), and the purified fragment was subcloned into the pGL3-basic vector (Promega). HKDC1 deletion reporter constructs were generated by 3-round PCR methods. Detailed information on these clones is available upon request (5).

### Establishment of Human PGC1β/HKDC1 Expression Cell Lines

The lentivirus for PGC1β expression was generated in our lab (5). The human HKDC1 cDNA was purchased from Open Biosystems and was subcloned into the pLVX-Puro vector (from Clontech). HKDC1 was amplified by below underlined primers with the introduction of Xho I and BamH I restriction sites: HKDC1 forward primer: 5′-ATCG-CTCGAG-atg ttt gcg gtc cac ttg atg-3′ (Xho I) and HKDC1 reverse primer: 5′-ATCG-GGATCC-cta gtt ctc ctt ctg tgc ctg-3′ (BamH I). The virus for HKDC1 or empty control (CTL) was expressed by Lenti-X™ Lentiviral Expression Systems (from Clontech). In order to establish stable human PGC1β/HKDC1 expression cell lines, the MCF7 or MDA231 cell line was infected by the lentivirus for either PGC1β, HKDC1, or empty control (CTL). The positive cells were selected by 10 μg/ml of puromycin, and the stable PGC1β or HKDC1 expression cell line was confirmed by real time PCR with more than 200% of mRNA increase compared to control group (see primers in Table S1) (5).

### Establishment of Stable PGC1β/HKDC1 Knockdown Cell Lines

The stable knockdown cells for PGC1β, HKDC1 or related non-target control (CTL) were prepared through infection of either MCF7 or MDA-MB-231 cell lines using shRNA lentivirus particles from Sigma for human PGC1β (SHCLNV-NM_133263), human HKDC1 (SHCLNV-NM_025130), or non-target control (SHC216V). The positive knockdown cells were selected by 10 μg/ml of puromycin, and the stable PGC1β or HKDC1 knockdown cell line was confirmed by real time PCR with more than 65% of mRNA decrease compared to control group (see primers in Table S1) (5).

### [^3^H]-deoxyglucose Uptake

1 × 10^6^ of treated cells were suspended and rinsed with PBS 3 times, then incubated with 1 ml of PBS containing 1.0 uCi ^3^H-deoxyglucose for 5 min at 37°C. Cells were washed with cold PBS 3 times, solubilized in 1 ml of 1 M NaOH for 60 min at 37°C, and then neutralized with an equal volume of 1 M HCl and counted in 10 ml scintillation mixture. The final results were normalized by protein level.

### Immunohistochemistry (IHC)

The CHTN BrCaProg1 Microarray slides were purchased from CHTN (Cooperative Human Tissue Network); the slide was firstly fixed by 3.7% formaldehyde solution, permeabilized by 1% BSA+0.2% Triton X-100 in PBS, then blotted by 40 μg/ml of rabbit antibody for either PGC1β or HKDC1 for 2 h. After another 1 h blotting by FITC labeled anti-rabbit secondary antibody, the slides were visualized and photographed, and the protein expression (60 cells in each group) were quantitated by Image J. software (5).

### Immunostaining

The treated cells were transferred to cover slips, and firstly stained by MitoTracker Red (#PA-3017 from Lonza, in option). The cells were then fixed by 3.7% formaldehyde solution, and stained by either Ki-67 (MIB-1) or HKDC1, and the nuclei of cells were stained by 4′,6-diamidino-2-phenylindole dihydrochloride (DAPI, #D9542, from Sigma), then the positive Ki-67 cells were quantitated (5, 28).

### Animals

The Balb/c athymic nude male mice (6 weeks old) were purchased from the Guangdong Medical Animal Center. The animal protocol was conducted in accordance with NIH regulations and were approved by the Institutional Animal Care and Use Committee (from Peking University Shenzhen Hospital). The detailed animal procedures was conducted as previously described (5, 28, 29).

### Methods

The mRNA was measured by real time quantitative PCR (5, 29, 30), the protein expression was measured by western blotting (WB) using the ODYSSEY Infrared Imaging System (LI-COR, NE), and the protein-protein interaction was evaluated by Immunoprecipitation (IP) (31). The Luciferase reporter assay was conducted using the Dual-Luciferase^TM^ Assay System (Promega) (28, 29). The binding ability of transcription factors on HKDC1 promoter was evaluated by chromatin immunoprecipitation (ChIP), and the precipitated DNA was then amplified by real-time PCR (qPCR) using the primers provided in Table S1 (28–30). The ROS generation was measured by CM-H2DCFDA (Invitrogen) using a FLx800 microplate fluorescence reader (Bio-Tek) (5, 29, 32). The apoptosis was evaluated by TUNEL assay using the *in situ* Cell Death Detection Kit™ (Roche), and the caspase-3 activity was determined by the ApoAlert caspase assay kit (Clontech), and the enzyme activity was measured using a FLx800 microplate reader (Bio-Tek) (5, 32). The mitochondrial function was evaluated by mitochondrial DNA copies (5, 30, 32), intracellular ATP level (5, 30, 32) and mitochondria membrane potential (5, 29, 33). The cell proliferation was evaluated by [^3^H]-deoxyglucose uptake, DNA synthesis by [^3^H]-thymidine incorporation (29), colony formation in soft agar (29), migration, and invasion assays (5, 34, 35). *In vivo* superoxide release was measured by a luminol-EDTA-Fe enhanced chemiluminescence (CL) system supplemented with DMSO-TBAC solution (32). The statistical analysis was conducted using SPSS 22 software and a *P*-value <0.05 was considered significant (29).

## Results

### Increased HKDC1 Expression Is Regulated by PGC1β in Breast Cancer Cells

**Figure 1:**
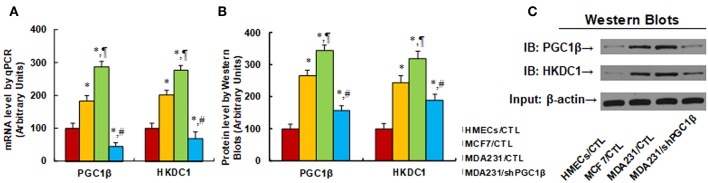
Increased HKDC1 expression is regulated by PGC1β in breast cancer cells. Different cells, including primary HMECs, MCF7 and MDA231 cells, were infected by either empty (CTL) or shPGC1β lentivirus, and the cells were harvested for gene expression analysis. **(A)** mRNA level by qPCR, *n* = 4. **(B)** Quantitation of protein levels, *n* = 5. **(C)** Representative pictures for Western Blotting. ^*^, *P* < 0.05, vs. HMECs/CTL group; ¶, *P* < 0.05 vs. MCF7/CTL group; #, *P* < 0.05, vs. MDA231/CTL group. Results are expressed as mean ± SEM.

We first evaluated the gene expression of HKDC1 and PGC1β in different breast cancer cells. In Figure 1A, the primary HMECs, breast tumor MCF7 cells, and breast cancer metastatic MDA231 cells were used for mRNA analysis. The results showed thatPGC1β mRNA levels increased to 184 and 287% in MCF7 and MDA231 cells, respectively, compared to HMECs, and PGC1β knockdown (shPGC1β) in MDA231 cells (MDA231/shPGC1β) decreased PGC1β mRNA to 16% compared to MDA231 control cells (MDA231/CTL). On the other hand, HKDC1 mRNA was increased to 201 and 276% in MCF7 and MDA231 cells, respectively, compared to HMECs, and the PGC1β knockdown (MDA231/shPGC1β) decreased HKDC1 mRNA to 25% compared to MDA231 control cells. We then evaluated the protein levels in those cells (see Figures 1B,C). The results showed that PGC1β protein levels increased to 265 and 345% in MCF7 and MDA231 cells, respectively, compared to HMECs, and the PGC1β knockdown (MDA231/shPGC1β) decreased PGC1β protein levels to 45% compared to MDA231 control cells (MDA231/CTL). On the other hand, HKDC1 protein levels increased to 245 and 319% in MCF7 and MDA231 cells, respectively, compared to HMECs, and the PGC1β knockdown (MDA231/shPGC1β) decreased HKDC1 protein to 59% compared to MDA231 control cells. We also measured mRNA levels for another two isoforms of the PGC1 family, including PGC1α and PPRC1 (36, 37), and found that there was no difference in those cells (see Figure S1a). We finally measured the mRNA levels for another 4 isoforms of hexokinase, including hexokinase 1 (HK1), HK2, HK3, and HK4 (see Figure S1b) (38, 39). The results showed that there was no difference in the cells for HK1, HK3, and HK4, while for HK2 (40), mRNA levels increased to 165 and 213% in MCF7 and MDA231 cells, respectively, compared to HMECs, while the PGC1β knockdown (MDA231/shPGC1β) did not change HK2 mRNA compared to MDA231 control cells. This means that even though HK2 expression increased in breast cancer cells, it is not regulated by PGC1β, indicating that another factor may contribute to its expression. Our results show that the expression of both PGC1β and HKDC1 increases in breast cancer cells, and the HKDC1 expression is regulated by PGC1β.

### HKDC1 Expression Is Regulated by PGC1β/SREBP1- Mediated Co-activation on the HKDC1 Promoter

**Figure 2:**
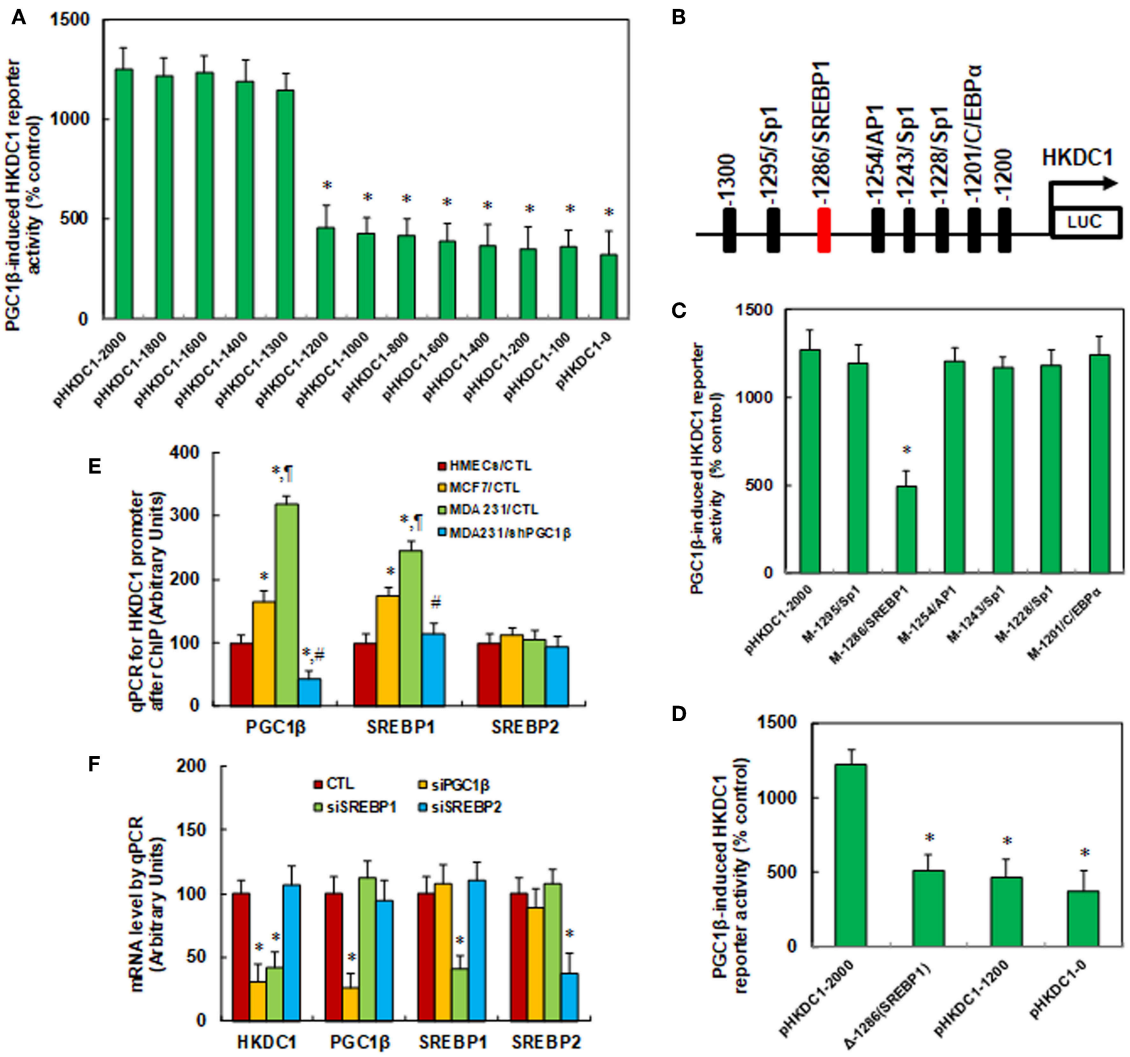
HKDC1 expression is regulated by PGC1β/SREBP1-mediated co-activation on the HKDC1 promoter. **(A)** The MCF7 cells were infected by either PGC1β (↑PGC1β) or empty control (CTL) lentivirus for 2 days, and then the cells were transiently transfected by either HKDC1 full length (pLDHA-2000) or deletion reporter plasmids. After 24 h, the PGC1β-induced HKDC1 reporter activities from PGC1β lentivirus infected cells (↑PGC1β) were calculated as the relative percentage (% control) by comparing to lentivirus empty control (CTL) infected cells. *, *P* < 0.05, vs. pHKDC1-2000 group, *n* = 5. **(B)** The schematic picture for the potential transcriptional binding motif in the range of−1300~-1200 (from transcription start site) on the HKDC1 promoter, and the potential SREBP1 binding site was marked in red. **(C)** The lentivirus-infected MCF7 cells were transiently transfected by either HKDC1 full length (pHKDC1-2000) or the specific transcriptional binding motif mutation reporter plasmids, and then the reporter activities were measured after 24 h. *, *P* < 0.05, vs. pHKDC1-2000 group, *n* = 5. **(D)** The lentivirus infected MCF7 cells were transiently transfected by HKDC1 full length (pHKDC1-2000) or truncate (pHKDC1-1200 and pHKDC1-0) reporters, or SREBP1 (located at the site of−1286) deletion plasmid [Δ-1286(SREBP1)], and after 24 h, the reporter activities were measured. *, *P* < 0.05, vs. pHKDC1-2000 group, *n* = 5. **(E)** Different breast cancer cells were used for ChIP analysis by PGC1β, SREBP1, or SREBP2 antibody, respectively, and the HKDC1 promoter in the range of−1300~-1100 was amplified and measured by qPCR, *n* = 5. *, *P* < 0.05, vs. HMECs/CTL group; ¶, *P* < 0.05 vs. MCF7/CTL group; #, *P* < 0.05, vs. MDA231/CTL group. **(F)** The MCF7 cells were transfected by siRNA for either non-sense control (CTL), PGC1β, SREBP1, or SREBP2 for 2 days, and the cells were harvested for mRNA analysis. *, *P* < 0.05, vs. CTL group, *n* = 5. Results are expressed as mean ± SEM.

We investigated the potential molecular mechanism for PGC1β-mediated HKDC1 expression in MCF7 cells. A series of progressive 5′-promoter deletion constructs for the HKDC1 promoter was generated, and these constructs were transfected into MCF7 cells for the reporter activity assay. We found that PGC1β-induced reporter activities were not markedly changed among the−2000,−1800,−1600,−1400, and−1300 deletion constructs (numbered according to Ensembl Transcript ID: ENST00000354624.5, transcription start site was marked as 0). However, the activity was reduced to 36% in the pHKDC1-1200 deletion reporter construct compared to the full length HKDC1 reporter (pHKDc1-2000), indicating that PGC1β-responsive transcriptional element is located in the range of−1300~-1200 on the HKDC1 promoter (see Figure 2A). The transcription factor databases TESS revealed several potential binding motifs, including a SREBP1 site located at−1286, marked in red (see Figure 2B). We then mutated these potential binding motifs, including C/EBPα site at−1201, 3 of Sp1 sites at−1228,−1243, and−1295, AP1 site at−1254 and SREBP1 site at−1286. The HKDC1 mutation reporter assay showed that mutation of SREBP1 binding motif at−1286 significantly decreased PGC1β-induced HKDC1 reporter activity compared to wild type HKDC1 full length (pHKDc1-2000) reporter (see Figure 2C). We further deleted the SREBP1 binding motif at−1286 [Δ-1286(SREBP1)] for the HKDC1 reporter activity assay (see Figure 2D). The results showed that SREBP1 deletion [Δ-1286(SREBP1)] reporter activity was decreased to 42% compared to pHKDC1-2000 full length reporter, while there was no significant difference compared to HKDC1 truncate reporters (pHKDC1-1200 and pHKDC1-0), indicating that SREBP1 binding motif at−1286 [-1286(SREBP1)] is required for PGC1β-induced HKDC1 activation. We also measured the binding abilities of PGC1β, SREBP1 and SREBP2 on the HKDC1 promoter using the ChIP technique in different breast cancer cells (see Figure 2E). The results showed that the binding ability of PGC1β and SREBP1 on the HKDC1 promoter was significantly increased to 165 and 174%, respectively, in MCF7, and increased to 318 and 245%, respectively, in MDA231 cells. Furthermore, the binding ability of PGC1β and SREBP1 on the HKDC1 promoter was decreased to 14 and 47%, respectively, as a result of PGC1β knockdown in MDA231 cells. On the other hand, the binding ability of SREBP2 on the HKDC1 promoter showed no difference, indicating that PGC1β and SREBP1 may bind to the HKDC1 promoter and be responsible for the HKDC1 activation, while SREBP2 has no effect. Finally, the siRNA technique was employed to knockdown the related transcription factors to evaluate their potential contributions on HKDC1 expression. We found that PGC1β knockdown (siPGC1β) decreased expression of HKDC1 and PGC1β to 31 and 26%, respectively, in MCF7 cells, while SREBP1 knockdown (siSREBP1) decreased expression of HKDC1 and SREBP1 to 42 and 41%, respectively, but had no effect on PGC1β. In addition, SREBP2 knockdown (siSREBP2) reduced SREBP2 expression to 37%, but had no effect on PGC1β, HKDC1, or SREBP1. Our results indicate that PGC1β and SREBP1 contribute to HKDC1 expression, while SREBP2 has no effect (see Figure 2F) (5).

### HKDC1 Expression Modulates Oxidative Stress, Apoptosis, and Mitochondrial Function in MCF7 Cells

**Figure 3:**
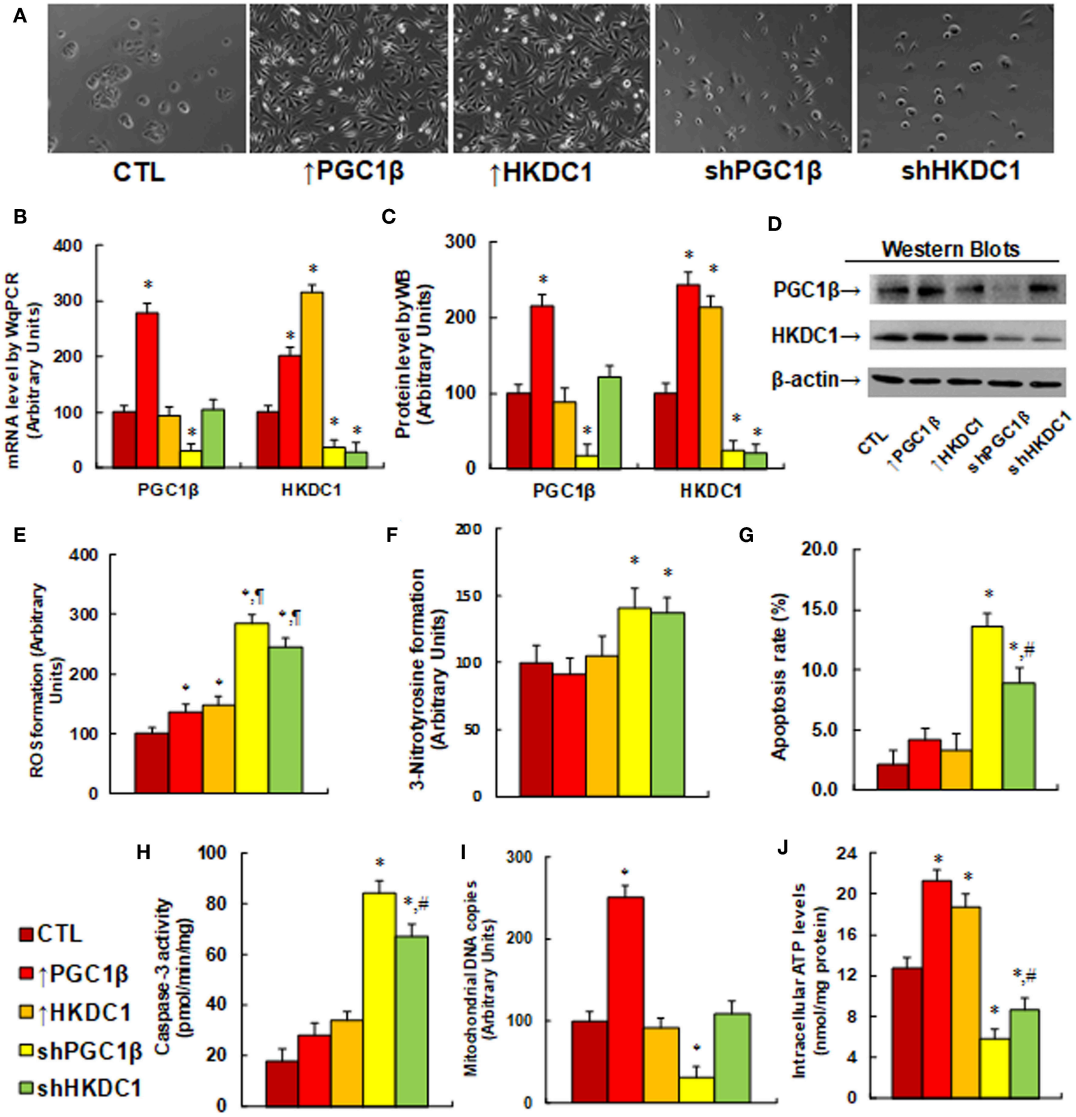
HKDC1 expression modulates oxidative stress, apoptosis and mitochondrial function in MCF7 cells. The MCF7 cells were infected by either expression or knockdown lentivirus for either PGC1β or HKDC1, and the subsequent stable cell lines or related empty vector control (CTL) were cultured for 2 days, and then the cells were harvested for further analysis. **(A)** Representative pictures for the morphological changes of treated MCF7 cells. **(B)** mRNA level by qPCR, *n* = 4. **(C)** Quantitation of protein levels, *n* = 5. **(D)** Representative pictures for Western Blotting. **(E)** ROS formation, *n* = 5. **(F)** 3-Nitrotyrosine formation, *n* = 5. **(G)** Apoptosis rate by TUNEL assay, *n* = 5. **(H)** Caspase-3 activity, *n* = 5. **(I)** Mitochondrial DNA copies, *n* = 4. **(J)** Intracellular ATP level, *n* = 5. *, *P* < 0.05, vs. CTL group; ¶, *P* < 0.05, vs. ↑PGC1β group; #, *P* < 0.05, vs. shPGC1β group. Results are expressed as mean ± SEM.

We evaluated the potential effect and molecular consequences of PGC1β/HKDC1 expression on MCF7 cells. PGC1β or HKDC1 was either overexpressed or knocked down by a lentivirus vector. We first evaluated the morphological changes of the cells after lentivirus-mediated gene expression manipulation. In Figure 3A, cells with overexpression of either PGC1β (↑PGC1β) or HKDC1 (↑HKDC1) significantly changed their cell shape, grew very quickly, and became into more metastatic style. On the other hand, knockdown of either PGC1β or HKDC1 caused the cells to grow very slowly, switch into a primary cell style, and lose their colony formation ability. We then measured the gene expression of the cells. In Figure 3B, PGC1β overexpression (↑PGC1β) increased mRNA of PGC1β and HKDC1 to 278 and 201%, respectively; HKDC1 overexpression (↑HKDC1) increased HKDC1 mRNA levels to 315%, but had no effect on PGC1β levels. On the other hand, PGC1β knockdown (shPGC1β) decreased mRNA levels of PGC1β and HKDC1 to 31 and 36%, respectively, compared to the control (CTL) group; HKDC1 knockdown (shHKDC1) decreased HKDC1 mRNA level to 29%, but had no effect on PGC1β. We then measured the protein levels of PGC1β and HKDC1 on the cells (see Figures 3C,D). We found that the protein levels showed patterns similar to that of the mRNA levels. Our results show that lentivirus-mediated PGC1β/HKDC1 expression manipulation was successful and efficient, and that PGC1β regulates HKDC1 expression, while HKDC1 does not affect PGC1β expression, indicating that HKDC1 is the downstream target gene of PGC1β. We then measured the potential effect of HKDC1 expression on oxidative stress (41). In Figure 3E, overexpression of PGC1β (↑PGC1β) and HKDC1 (↑HKDC1) slightly increased ROS formation to 136 and 148%, respectively, compared to the control (CTL) group. On the other hand, knockdown of PGC1β (shPGC1β) and HKDC1 (shHKDC1) significantly increased ROS formation to 285 and 245%, respectively, and the effect of shHKDC1 had a smaller effect than that of shPGC1β treatment. We also measured 3-nitrotyrosine formation (see Figure 3F). The results showed that expression of PGC1β and HKDC1 had no effect, while knockdown of PGC1β and HKDC1 increased 3-nitrotyrosine formation to 141 and 137%, respectively, compared to CTL group. Our results suggest that HKDC1 downregulation may result in significant cytotoxicity due to oxidative stress. We then measured apoptosis rate and caspase-3 activity. The results showed that expression of PGC1β and HKDC1 had no effect, while knockdown of PGC1β (shPGC1β) and HKDC1 (shHKDC1) increased apoptosis rates to 648 and 424%, respectively, compared to CTL group (see Figure 3G), and increased caspase-3 activity to 467 and 372%, respectively (see Figure 3H). Furthermore, shHKDC1 had a significantly smaller effect compared to shPGC1β treatment. We then measured mitochondrial DNA copies (see Figure 3I). The results showed that PGC1β expression increased while PGC1β knockdown decreased mitochondrial DNA copies to 251 and 31%, respectively, compared to CTL group. On the other hand, HKDC1 had no effect on mitochondrial DNA copies. Our results suggest that mitochondrial DNA replication may be regulated by PGC1β instead of HKDC1. We finally evaluated the effect of gene expression on intracellular ATP levels (see Figure 3J). Our results showed that overexpression of PGC1β (↑PGC1β) and HKDC1 (↑HKDC1) significantly increased ATP generation to 168 and 147%, respectively, compared to CTL group. On the other hand, knockdown of PGC1β (shPGC1β) and HKDC1 (shHKDC1) significantly decreased ATP generation to 46 and 68%, respectively, and shHKDC1 treatment had a smaller effect than shPGC1β treatment. Our results show that HKDC1 expression modulates oxidative stress, apoptosis, and mitochondrial function in breast cancer cells.

### HKDC1 Expression Modulates Permeability Transition Pore and Glucose Uptake by Binding With VDAC1 in MCF7 Cells

**Figure 4:**
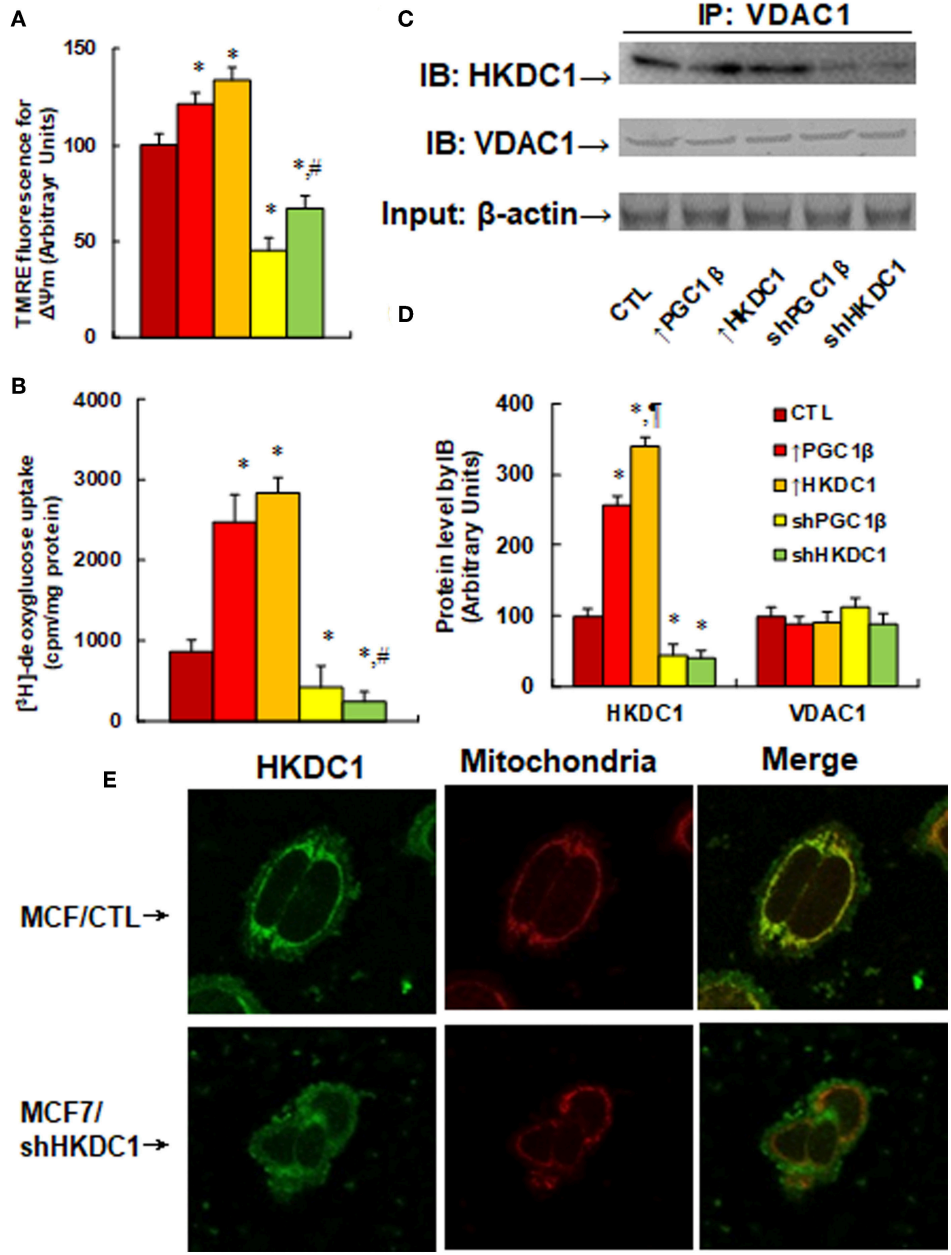
HKDC1 expression modulates permeability transition pore and glucose uptake by binding with VDAC1 in MCF7 cells. The MCF7 cells were infected by either expression or knockdown lentivirus for either PGC1β or HKDC1, and the subsequent stable cell lines or related empty vector control (CTL) were cultured for 2 days, then the cells were harvested for further analysis. **(A)** Mitochondrial membrane potential (ΔΨm), *n* = 5. **(B)** [^3^H]-deoxyglucose uptake, *n* = 4. **(C)** The treated cells were IP by VDAC1, then IB by HKDC1, and VDAC1, with 10% of β-actin from whole lysates as input control. **(D)** Protein quantitation for **(C)**, *n* = 4. **(E)** The MCF7 cells were infected by either lentivirus empty control (CTL) or shHKDC1, then the cells were immunostained by HKDC1 (green), and the mitochondria was stained by MitoTracker Red (red). *, *P* < 0.05, vs. CTL group; ¶, *P* < 0.05, vs. ↑PGC1β group; #, *P* < 0.05, vs. shPGC1β group. Results are expressed as mean ± SEM.

We first measured the effects of HKDC1 expression on the mitochondrial membrane potential (MMP, ΔΨm) (see Figure 4A). The results showed that overexpression of PGC1β and HKDC1 slightly increased MMP to 121 and 134%, respectively, compared to CTL group, while knockdown of PGC1β and HKDC1 significantly decreased MMP to 45 and 67%, respectively, and the effect of shHKDC1 has less of an effect than shPGC1β. We then measured [^3^H]-deoxyglucose uptake (see Figure 4B). The results showed that overexpression of PGC1β and HKDC1 significantly increased glucose uptake to 286 and 328%, respectively, compared to CTL group, while knockdown of PGC1β and HKDC1 decreased glucose uptake to 50 and 27%, respectively, with shHKDC1 having a stronger effect than shPGC1β. We also measured the interaction of HKDC1 with VDAC1 using IP/WB techniques (see Figures 4C,D). The results showed that overexpression of PGC1β and HKDC1 significantly increased interaction of HKDC1 with VDAC1 to 256 and 341%, respectively, compared to CTL group, while knockdown of PGC1β and HKDC1 decreased interaction to 45 and 39%, respectively. Furthermore, the VDAC1 protein level had no change in different treatments, so the precipitated HKDC1 levels reflected the protein levels of HKDC1. We finally investigated the cellular location of HKDC1 using immunostaining techniques (see Figure 4E). We found that HKDC1 (stained with green) had colocalization with mitochondria (stained with red) in wild type MCF7 cells, while shHKDC1 cells showed significant loss of HKDC1 on the mitochondria. Our results indicate that HKDC1 is located on the mitochondria membrane, interacts with VDAC1, and modulates the permeability transition pore, subsequently modulating glucose uptake in breast cancer cells.

### HKDC1 Expression Potentiates Cell Proliferation, While HKDC1 Knockdown Reverses This Effect in MCF7 Cells

**Figure 5:**
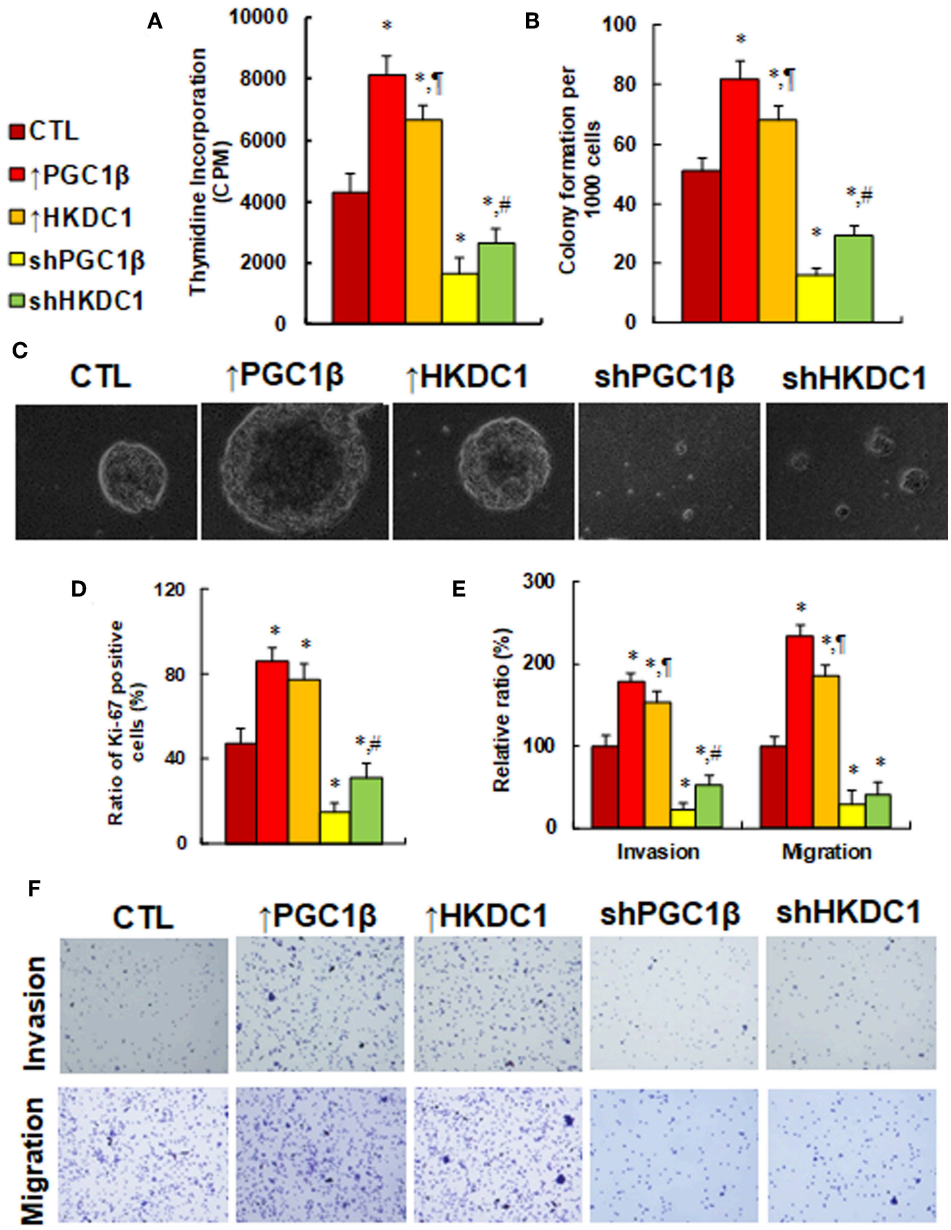
HKDC1 expression potentiates cell proliferation, while HKDC1 knockdown reverses this effect in MCF7 cells. The MCF7 cells were infected by either expression or knockdown lentivirus for either PGC1β or HKDC1, and the subsequent stable cell lines or related empty vector control (CTL) were cultured for 2 days, and then the cells were harvested for further analysis. **(A)** Cell proliferation analysis by thymidine incorporation, *n* = 5. **(B)** Colony formation assay in soft agar, *n* = 5. **(C)** Representative pictures for **(B)**. **(D)** Quantitation of Ki-67 positive cells, *n* = 3. **(E)** Cell invasion and migration assay, *n* = 4. **(F)** Representative picture for **(E)**. *, *P* < 0.05, vs. CTL group; ¶, *P* < 0.05, vs. ↑PGC1β group; #, *P* < 0.05, vs. shPGC1β group. Results are expressed as mean ± SEM.

We investigated the potential effect of HKDC1 expression on *in vitro* cell proliferation in MCF7 cells. In Figure 5A, cell proliferation was evaluated by thymidine incorporation. The results showed that overexpression of PGC1β (↑PGC1β) and HKDC1 (↑HKDC1) increased thymidine incorporation to 189 and 155%, respectively, compared to control (CTL) group, while knockdown of PGC1β (shPGC1β) and HKDC1 (shHKDC1) decreased thymidine incorporation to 38 and 62%, respectively, and HKDC1 showed less of an effect than PGC1β. We then investigated *in vitro* colony formation in soft agar (see Figures 5B,C). The results showed that treatment of ↑PGC1β and ↑HKDC1 increased colony formation to 161 and 133%, respectively, compared to CTL group. On the other hand, treatment of shPGC1β and shHKDC1 decreased colony formation to 31 and 57%, respectively. We also measured the Ki-67 positive ratio using immunostaining techniques (see Figure 5D). We found that treatment of ↑PGC1β and ↑HKDC1 increased Ki-67 positive ratio to 183 and 164% respectively compared to CTL group, while treatment of shPGC1β and shHKDC1 decreased Ki-67 positive ratio to 32 and 66%, respectively. In addition, shHKDC1 had a significantly lesser effect than shPGC1β. We finally investigated the effect of HKDC1 on cell invasion and migration (see Figures 5E,F). The results showed that treatment of ↑PGC1β and ↑HKDC1 increased cell invasion to 179 and 154%, respectively, compared to CTL group, while treatment of shPGC1β and shHKDC1 decreased cell invasion to 22 and 53%, respectively. Furthermore, treatment of ↑PGC1β and ↑HKDC1 increased cell migration to 234 and 185%, respectively, compared to CTL group, while treatment of shPGC1β and shHKDC1 decreased cell migration to 29 and 41%, respectively. In addition, the effect of HKDC1 had a smaller effect than PGC1β. Our results show that HKDC1 expression modulates *in vitro* cell proliferation in MCF7 cells and that PGC1β has a stronger effect than HKDC1; this suggests that other factors mediated by PGC1β may also be involved in modulation.

### HKDC1 Expression Is Involved With Tumor Growth and Metastasis *in vivo*

**Figure 6:**
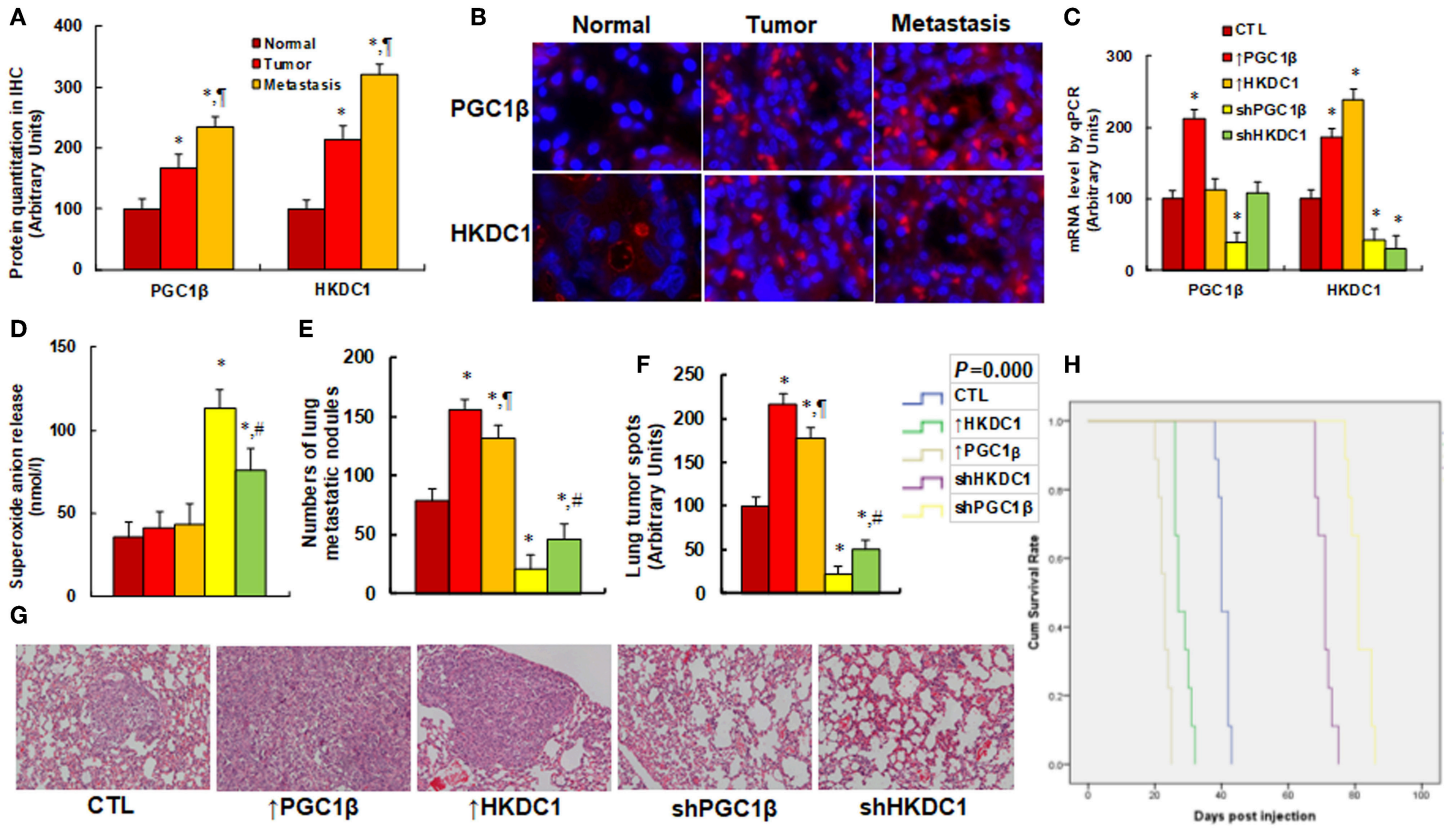
HKDC1 expression involves with tumor growth and metastasis *in vivo*. **(A,B)** Tissue Microarray slides (#CHTN BrCaProg1) were obtained from CHTN (Cooperative Human Tissue Network), and the immunohistochemistry was performed using a primary rabbit antibody for either PGC1β or HKDC1 and a second anti-rabbit- FITC. Sixty cells from normal breast tissue (normal), primary tumor (tumor), and metastatic (metastasis) tissues were quantitated by Image J. **(A)** Protein quantitation, *n* = 3. *, *P* < 0.05, vs. Normal group; ¶, *P* < 0.05 vs. Tumor group. **(B)** Representative pictures for **(A)**. **(C–H)** The nude mice were injected with treated MCF7 cells through the tail vein for *in vivo* xenograft tumor development study, and the treated mice were sacrificed for further analysis. **(C)** The tumor tissues from the lung were isolated for mRNA analysis by qPCR, *n* = 4. *, *P* < 0.05, vs. CTL group. **(D)** Superoxide anion release from tumor tissues, *n* = 5, *, *P* < 0.05, vs. CTL group; #, *P* < 0.05, vs. shPGC1β group. **(E–G)** Mice were killed upon 20% weight loss, and the lungs were harvested for terminal analysis. The metastatic tumor nodules from the lungs were counted, and then the formalin-fixed, paraffin-embedded tumor tissue of the lung was sectioned to 4 mm thickness, and the histopathological analyses were performed with H&E staining. Images were taken using a Carl Zeiss MIRAX MIDI slide scanner, and the lung tumor spots were analyzed using a 3DHISTECH Pannoramic Viewer. **(E)** Tumor colony formation in lung, *n* = 9. *, *P* < 0.05, vs. CTL group; ¶, *P* < 0.05, vs. ↑PGC1β group; #, *P* < 0.05, vs. shPGC1β group. **(F)** Quantitated lung tumor spots, *n* = 5. *, *P* < 0.05, vs. CTL group; ¶, *P* < 0.05, vs. ↑PGC1β group; #, *P* < 0.05, vs. shPGC1β group. **(G)** Representative picture by H&E staining. **(H)** Kaplan-Meier analysis comparing survival of mice between each treatment group, *P*-value represents log-rank Mantel-Cox test result, *n* = 9. Results are expressed as mean ± SEM.

We first evaluated the gene expression of PGC1β and HKDC1 in human breast cancer tissues. CHTN BrCaProg1 Microarray slides were obtained from CHTN, including normal, tumor, and metastatic breast cancer tissues. The slides were stained for HKDC1 and PGC1β using immunohistochemistry techniques (see Figures 6A,B). Our results showed that PGC1β protein expression in tumor and metastatic tissues was increased to 168 and 234%, respectively, compared to normal tissues, while the HKDC1 protein expression in tumor and metastatic tissues were increased to 214 and 321%, respectively, compared to normal tissues. This provides powerful evidence that the expression of PGC1β and HKDC1 is increased in human breast cancer tissues. We then investigated the potential effect of HKDC1 expression in the *in vivo* xenograft tumor development study using treated MCF7 cells. The nude mice were injected with MCF7 cells through the tail vein, subsequent xenograft tumor tissues from the lungs were isolated and analyzed, and the mouse survival rates were calculated (5). We first measured mRNA expression of PGC1β and HKDC1 from the tumor tissues (see Figure 6C). The results showed that ↑PGC1β treatment increased expression of PGC1β and HKDC1 to 234 and 168%, respectively, compared to CTL group, and ↑HKDC1 treatment increased HKDC1 expression to 215%, but showed no effect on PGC1β expression. Furthermore, shPGC1β treatment decreased expression of PGC1β and HKDC1 to 31 and 32%, respectively, compared to CTL group, and shKDC1 treatment decreased HKDC1 expression to 21%, but showed no effect on PGC1β expression. This indicates that lentivirus-mediated *in vivo* gene manipulation was efficient and that HKDC1 expression is regulated by PGC1β. We then evaluated superoxide anion (${\text{O}}_{2}^{-.}$) release from the xenograft tumor tissues. The results showed that ↑HKDC1 treatment slightly increased superoxide anion (${\text{O}}_{2}^{-.}$) release to 194% compared to CTL group, while ↑PGC1β treatment showed no effect. In addition, treatment of shPCG1β and shHKDC1 significantly increased superoxide anion (${\text{O}}_{2}^{-.}$) release in tumor tissues to 390 and 303%, respectively (see Figure 6D). We also investigated the effect of HKDC1 expression on lung tumor nodule formation. The results showed that treatment of ↑PGC1β and ↑HKDC1 increased tumor colony formation in the lung to 166 and 126%, respectively. On the other hand, knockdown of PGC1β and HKDC1 decreased colony formation to 29 and 38%, respectively (see Figure 6E). We then investigated the lung tumor spots using H&E staining (see Figures 6F,G). The results showed that treatment of ↑PGC1β and ↑HKDC1 increased lung tumor spots to 208 and 189%, respectively. On the other hand, treatment of shPGC1β and shHKDC1 decreased lung tumor spots to 31 or 56%, respectively. We eventually investigated the effect of HKDC1 expression on mouse survival rates using Kaplan-Meier analysis (see Figure 6H). The results showed that treatment of ↑PGC1β and ↑HKDC1 largely decreased mouse survival, while knockdown of PGC1β and HKDC1 significantly increased mouse survival. In addition, the PGC1β had a stronger effect than HKDC1 on *in vivo* tumor growth. We also briefly repeated the same experiments for *in vivo* xenograft tumor development study using treated MDA231 cells (see Figure S2), and results similar to those of MCF7 were observed. In Figure S2a, we measured mRNA levels for the expression of PGC1β and HKDC1. The results showed that manipulation of gene expression by lentivirus vector was successful and efficient, and that PGC1β regulates HKDC1, while HKDC1 does not affect PGC1β expression. In Figure S2b, we found that knockdown of either PGC1β or HKDC1 significantly increased superoxide anion release. Furthermore, the expression of PGC1β and HKDC1 significantly increased metastatic colony formation (see Figure S2c) and tumor spots in the lung (see Figure S2d). On the other hand, knockdown of PGC1β and HKDC1 significantly deceased tumor growth. Our results suggest that HKDC1 expression is involved with tumor growth and that this effect is modulated by PGC1β, and PGC1β also seems to be involved with some other factors (5).

## Discussion

In this study, we demonstrate that HKDC1 expression is increased significantly in breast cancer cells, and is regulated by PGC1β/SREBP1-mediated co-activation on the HKDC1 promoter. HKDC1 expression potentiates the permeability transition pore by interacting with VDAC1, subsequently increasing glucose uptake and promoting cell proliferation and tumor growth.

### HKDC1-Mediated Mitochondrial Function

HKDC1 is a newly discovered protein and its detailed cellular function remains largely unknown. Because HKDC1 sequences that are significantly identical to those of other hexokinase isoforms, we suggest that HKDC1 may have function similar to that of a hexokinase enzyme in the regulation of glucose phosphorylation and mitochondrial binding (15, 16, 19, 39). It has been reported that hexokinases interact with VDAC and directly couple intramitochondrial ATP synthesis to glucose metabolism (42, 43). Our results show that knockdown of PGC1β or HKDC1 increases ROS formation, 3-nitrotyrosine formation, apoptosis rate, and caspase-3 activity depending on the protein level of HKDC1, this can be explained that decreased HKDC1 expression dissociates HKDC1 from VDAC1, results in decreased mitochondrial membrane potential, subsequently triggers cell damage. On the other hand, overexpression of PGC1β or HKDC1 with increased HKDC1 protein level does not affect these factors, since it achieves increased binding of HKDC1 with VDAC1, and subsequently increases glucose uptake with potentiated cell proliferation. Furthermore, when the HKDC1 is knocked down, HKDC1 has less interaction with mitochondria, and is not localized to mitochondria, this may due to HKDC1 knockdown changes the potential property of outer mitochondrial membrane. Our study has confirmed this effect in HKDC1, showing that HKDC1 binds with VDAC1, increases mitochondrial membrane potential, and subsequently increases glucose uptake (44). This suggests that the permeability transition pore opening is regulated by HKDC1 through interaction with VDAC1 at the outer mitochondrial membrane (45).

### PGC1β/SREBP1-Mediated Gene Regulation

SREBPs regulate cellular lipid metabolism and homeostasis (46), and SREBP-mediated lipogenic gene expression is regulated by PGC1β (10). Furthermore, SREBP1 is involved in tumorigenesis and related gene expression (9, 14, 47). In this study, we showed that HKDC1 is regulated by the PGC1β/SREBP1 signaling pathway through the SREBP1 binding motif located on the HKDC1 promoter. It increases the permeability transition pore opening by interacting with VDAC, subsequently increasing glucose uptake and promoting tumor cell proliferation and growth. This is the first time we report the potential role of SREBP1 in tumor growth, which is consistent with previous reports (48, 49). This provides us with a new anti-tumor strategy through targeting SREBP1 (50).

### HKDC1 as the New Target for Cancer Therapy

It has been reported that HKDC1 catalyzes glucose phosphorylation and the related cellular energy metabolism involves with cancer growth and metastasis (18, 51), although the potential role of HKDC1 in cancer development remains unclear. Recent bioinformatics analysis has shown that HKDC1 is a novel potential therapeutic target for cancer (22). In addition, HKDC1 expression increases in hepatocarcinoma and is associated with poor prognosis (18). In this study, we show that the expression of both HK2 (38) and HKDC1 is increased, and that the HKDC1 is regulated by PGC1β, while HK2 is not. Furthermore, our results show that HKDC1 knockdown has less of an effect than PGC1β knockdown on the suppression of tumor growth, indicating that PGC1β-induced tumor growth is not only mediated by HKDC1, but also by other factors, such as LDHA (5) and HK2 (38). This study indicates that HKDC1 may be a new target for cancer therapy. Our preliminary results show that HKDC1 contributes to both endocrine and chemo resistance, and the inhibition of HKDC1 activity by either chemical or biological method significantly suppresses the tumor growth, and this investigation is still in process.

### Conclusions

Taken together, the HKDC1 expression is increased significantly in breast cancer cells and tumor tissues, and is co-activated by PGC1β through the SREBP1 binding motif on the HKDC1 promoter. HKDC1 is located on the mitochondrial membrane and regulates the permeability transition pore opening by binding with VDAC1, subsequently modulating glucose uptake, cell proliferation, and tumor growth. This is the first time that the mechanism for PGC1β-mediated HKDC1 expression has been discovered in breast cancers, and this provides a new strategy for cancer therapy through targeting the PGC1β/HKDC1 signaling pathway.

## Ethics Statement

The animal protocol conformed to US NIH guidelines (Guide for the Care and Use of Laboratory Animals, No. 85–23, revised 1996), and was reviewed and approved by the Institutional Animal Care and Use Committee from Wuhan University.

## Author Contributions

PY wrote the paper. PY, ZY, and XH designed, interpreted the experiments. HZ, QC, and JF performed the HKDC1 plasmids construction and mapping experiments. LZ and LL performed the gene analysis. WX, ML, AY, and QZ performed gene analysis and mouse experiments. XC, YL, and YS performed the remaining experiments. All authors read, edited, and approved the final manuscript.

### Conflict of Interest Statement

The authors declare that the research was conducted in the absence of any commercial or financial relationships that could be construed as a potential conflict of interest.
