# Supplementary material for: PGC1β Regulates Breast Tumor Growth and Metastasis by SREBP1-Mediated HKDC1 Expression
Source: Front Oncol. 2019 Apr 17;9:290. doi: 10.3389/fonc.2019.00290 (PMC6478765; doi:10.3389/fonc.2019.00290)
Supplement: Supplementary file 1 [file Data_Sheet_1.docx]

**PGC1β Regulates Breast Tumor Growth and Metastasis by SREBP1-Mediated**

**HKDC1 Expression**

Xiaoli Chen^1,^*, Yang Lv^2,^*, Ying Sun^2,^*, Hongyu Zhang^3^, Weiguo Xie^1^, Liyan Zhong^2^,

Qi Chen^3^, Min Li^1^, Ling Li^2^, Jia Feng^3^, Athena Yao^1^, Qi Zhang^4^,

Xiaodong Huang^1,#^, Zhendong Yu^3,#^, Paul Yao^1,2,3,#^

**Supplemental Information**

**Data S1. Materials and Methods**

**Materials and Reagents**. Human primary mammalian epithelial cells (HMECs, obtained from Lonza) were cultured in MEGM BulletKit (CC-3150). MCF7 and MDA-MB-231 (MDA231, obtained from ATCC) were cultured in DMEM at 37˚C supplemented with 10% FBS and antibiotics.

Antibodies for β-actin (sc-47778), Ki-67 (sc-101861), SREBP1 (sc-13551), SREBP2 (sc-13552) and VDAC1 (sc-390996) were obtained from Santa Cruz Biotechnology. Antibodies for HKDC1 (ab228729) and PGC1β (ab176328) were obtained from Abcam. 3-nitrotyrosine (3-NT) was measured using the 3-Nitrotyrosine ELISA Kit (ab116691 from Abcam). The Coomassie Protein Assay Kit (Pierce Biotechnology) was used to measure the protein concentration. The siRNA for SREBP1, SREBP2, PGC1β and negative control (#AM4636) were purchased from Ambion. The Lipofectamine™ Reagent (Invitrogen) was used for DNA transfection (1).

**Construction of HKDC1 reporter plasmids**. The HKDC1 promoter (2000bp upstream of TSS + first exon) from the Ensembl Transcription ID: HKDC1-201 (ENST00000354624.5) was amplified from human genomic DNA in HMEC cells by PCR using the following primers with the introduction of *Kpn* I/*Hind III* restriction sites as indicated by underline, HKDC1 Forward: 5’- gcgc- GGTACC - gaa aag gat ggg gat cct caa - 3’ (*Kpn* I) and HKDC1 Reverse: 5’- gcgc- AAGCTT- ctt ctt gat ctg gtc ctc ctt - 3’(*Hind III*), and the purified fragment was subcloned into the pGL3-basic vector (Promega). HKDC1 deletion reporter constructs were generated by 3-round PCR methods. Detailed information on these clones is available upon request (1).

**Establishment of human PGC1β/HKDC1 expression cell lines.** The lentivirus for PGC1β expression was generated in our lab (1). The human HKDC1 cDNA was purchased from Open Biosystems and was subcloned into the pLVX-Puro vector (from Clontech). HKDC1 was amplified by below underlined primers with the introduction of Xho I and BamH I restriction sites: HKDC1 forward primer: 5’- ATCG- CTCGAG- atg ttt gcg gtc cac ttg atg -3’ (Xho I) and HKDC1 reverse primer: 5’- ATCG- GGATCC- cta gtt ctc ctt ctg tgc ctg - 3’ (BamH I). The virus for HKDC1 or empty control (CTL) was expressed by Lenti-X™ Lentiviral Expression Systems (from Clontech). In order to establish stable human PGC1β/HKDC1 expression cell lines, the MCF7 or MDA231 cell line was infected by the lentivirus for either PGC1β, HKDC1 or empty control (CTL). The positive cells were selected by 10μg/ml of puromycin, and the stable PGC1β or HKDC1 expression cell line was confirmed by real time PCR with more than 200% of mRNA increase compared to control group (see primers in Table S1) (1).

**Establishment of stable PGC1β/HKDC1 knockdown cell lines**. The stable knockdown cells for PGC1β, HKDC1 or related non-target control (CTL) were prepared through infection of either MCF7 or MDA-MB-231 cell lines using shRNA lentivirus particles from Sigma for human PGC1β (SHCLNV-NM_133263), human HKDC1 (SHCLNV-NM_025130), or non-target control (SHC216V). The positive knockdown cells were selected by 10μg/ml of puromycin, and the stable PGC1β or HKDC1 knockdown cell line was confirmed by real time PCR with more than 65% of mRNA decrease compared to control group (see primers in Table S1) (1).

**RT reaction and real-time quantitative PCR.** The total RNA was extracted by RNeasy Micro Kit (Qiagen), and was reverse transcribed by an Omniscript RT kit (Qiagen). All the primers were designed by Primer 3 Plus software (see Table S1), and the amplified products were confirmed by agarose gel. Real-time quantitative PCR was run on iCycler iQ (Bio-Rad) with the Quantitect SYBR green PCR kit (Qiagen). The PCR was performed by denaturing at 95°C for 8 min, followed by 45 cycles of denaturation at 95°C, annealing at 60°C, and extension at 72°C for 10s, respectively. 1 µl of each cDNA was used to measure target genes. β-actin was used as the housekeeping gene for transcript normalization, and the mean values were used to calculate relative transcript levels with the ^ΔΔ^CT method per instructions from Qiagen. In brief, the amplified transcripts were quantified by the comparative threshold cycle method using β-actin as a normalizer. Fold changes in gene mRNA expression were calculated as 2^−ΔΔCT^ with CT = threshold cycle, ΔCT=CT (target gene)-CT(β-actin), and the ΔΔCT =ΔCT (experimental)-ΔCT (reference) (1-3).

Immunoprecipitation (IP) and Western Blotting (WB). Cell lysates were pre-cleared by pre-immune IgG plus Protein A agarose beads for 2 hours, and the supernatants were immunoprecipitated by the indicated antibodies and a 50% slurry of Protein A Agarose beads overnight at 4˚C (4). After washing with buffer containing 50 mM Tris, pH 7.5, 150 mM NaCl, 1% NP-40, and 0.5% deoxycholate with protease inhibitors, proteins were released, separated on 10% SDS-PAGE gels, blotted by primary antibodies, then simultaneously incubated with the differentially labeled species-specific secondary antibodies, anti-RABBIT IRDye™ 800CW (green) and anti-MOUSE (or goat) ALEXA680 (red). Membranes were scanned and quantitated by the ODYSSEY Infrared Imaging System (LI-COR, NE) (5).

**Luciferase reporter assay.** 1.0×10^5^ of cells were seeded in a 6-well plate with complete medium to grow until they reached 80% confluence. Cells were then cotransfected by 3µg of HKDC1 full length or deletion reporter constructs, together with 0.2µg of pRL-CMV-Luc *Renilla* plasmid (from Promega) for 24 hours. After treatment, the cells were harvested and the luciferase activity assays were carried out using the Dual-Luciferase^TM^ Assay System (Promega). The transfection efficiencies were normalized using a cotransfected *Renilla* plasmid according to manufacturers’ instructions, and the HKDC1 reporter activity was calculated (3, 6).

**Chromatin Immunoprecipitation (ChIP).** Cells were washed and crosslinked using 1% formaldehyde for 20 min and terminated by 0.1M glycine. Cell lysates were sonicated and centrifuged. 500µg of protein were pre-cleared by BSA/salmon sperm DNA with preimmune IgG and a slurry of Protein A Agarose beads. Immunoprecipitations were performed with the indicated antibodies, BSA/salmon sperm DNA and a 50% slurry of Protein A agarose beads. Input and immunoprecipitates were washed and eluted, then incubated with 0.2mg/ml Proteinase K for 2h at 42˚C, followed by 6h at 65˚C to reverse the formaldehyde crosslinking. DNA fragments were recovered through phenol/chloroform extraction and ethanol precipitation. A ~150bp fragment in the range of -1400~-1100 from the transcription start site on the HKDC1 promoter was amplified by real-time PCR (qPCR) using the primers provided in Table S1 (2, 3, 6).

**Measurement of ROS generation.** Treated cells were seeded in a 24-well plate and incubated with 10μM CM-H2DCFDA (Invitrogen) for 45 min at 37°C, and then the intracellular formation of reactive oxygen species (ROS) was measured at excitation/emission wavelengths of 485/530nm using a FLx800 microplate fluorescence reader (Bio-Tek). The data was normalized as arbitrary units (3, 7) (1).

**Measurement of apoptosis.** Apoptosis was evaluated by TUNEL assay using the In Situ Cell Death Detection Kit™ (Roche). Cells were fixed in 4% paraformaldehyde and labeled by TUNEL reagents. Stained cells were photographed by a fluorescence microscope and further quantified by FACS analysis. Caspase-3 activity was determined by the ApoAlert caspase assay kit (Clontech). Treated cells were harvested and 50 µg of proteins were incubated with the fluorogenic peptide substrate Ac-DEVD-7-amino-4-trifluoromethyl coumarin (AFC). The initial rate of free AFC release was measured using a FLx800 microplate reader (Bio-Tek) at excitation/emission wavelengths of 380/505nm, and enzyme activity was calculated as pmol/min/mg (1, 7).

**Measurement of mitochondrial function.**

*Mitochondrial DNA copies.* Genomic DNA was extracted from treated MM.1R cells using a QIAamp DNA Mini Kit (Qiagen) and the mitochondrial DNA was extracted using the REPLI-g Mitochondrial DNA Kit (Qiagen). The purified DNA was used for the analysis of genomic β-actin (marker of the nuclear gene) and ATP6 (ATP synthase F0 subunit 6, marker of the mitochondrial gene) respectively using the qPCR method as mentioned above. The primers for genomic β-actin: forward 5’- ctg gac ttc gag caa gag atg -3’ and reverse: 5’- agg aag gaa ggc tgg aag agt -3’. The primers for ATP6: forward 5’- cat tta cac caa cca ccc aac -3’ and reverse 5’- tat ggg gat aag ggg tgt agg -3’. The mitochondrial DNA copies were obtained from relative ATP6 copies that were normalized by β-actin copies using the ^ΔΔ^CT method (1, 2, 7).

*Intracellular ATP level.* The intracellular ATP level was determined using the luciferin/luciferase-induced bioluminescence system. An ATP standard curve was generated at concentrations of 10^-12^-10^-3^M. Intracellular ATP levels were calculated and expressed as nmol/mg protein (1, 2, 7).

*Mitochondria membrane potential*. The mitochondrial membrane potential (MMP, Δψm) was measured using TMRE (from Molecular Probes T-669) staining. A 600μM T-669 stock solution was prepared using DMSO. Cells were grown on coverslips and immersed in 600nM TMRE for 20 min at 37°C to load them with dye. The labeling medium was then aspirated and the cells were immersed in 150nM TMRE to maintain the equilibrium distribution of the fluorophore. The coverslips were mounted with live cells onto confocal microscopes to image the cells using 548nm excitation/573nm emission filters. The intensity of TMRE fluorescence was measured using Image J software. Data from 10-20 cells were collected for each experimental condition and mean values of fluorescence intensity ± SEM were calculated (1, 3, 8).

**[^3^H]-deoxyglucose uptake.** 1x10^6^ of treated cells were suspended and rinsed with PBS 3 times, then incubated with 1ml of PBS containing 1.0uCi ^3^H-deoxyglucose for 5 min at 37°C. Cells were washed with cold PBS 3 times, solubilized in 1ml of 1M NaOH for 60min at 37°C, and then neutralized with an equal volume of 1 M HCl and counted in 10ml scintillation mixture. The final results were normalized by protein level.

**DNA synthesis by [^3^H]-thymidine incorporation**. Cell proliferation was evaluated as the rate of DNA synthesis by [^3^H]-methylthymidine incorporation (9). Cells were pooled in 24-well plates until they reached 80% confluence, and then the indicated chemicals were added and incubated for 24 hours. At the end of the treatment, cells were incubated with serum-free media containing ^3^H-methylthymidine (0.5 µCi/well) for 2 hours and then washed twice with PBS. Cellular DNA was precipitated by 10% trichloroacetic acid and solubilized with 0.4M NaOH (0.5 ml/well). Incorporation of ^3^H-methylthymidine into DNA was measured in a scintillation counter and was determined as counts per minute (CPM) (3).

**Colony formation in soft agar**. This assay is a method for evaluating the ability of individual cell lines to grow in an anchorage-independent manner. Cells were resuspended in DMEM containing 5% FBS with 0.3% agarose and layered on top of 0.5% agarose in DMEM on 60-mm plates. 1000 cells were seeded in 60mm soft agar dishes for 30 days. The dishes were examined twice per week; colonies that grew beyond 50mm in diameter were scored as positive, and the representative pictures were taken. Each experiment was done in quadruplicate (3).

**Migration and invasion assays**. Cell migration and invasion assays were performed in 24-well chemotaxis plates with an 8μm polycarbonate filter membrane, uncoated for migration assays or coated with 20μg Matrigel for invasion assays. Invasion or migration was expressed as the number of migrated cells bound per microscopic field and averaged from at least four fields per assay in at least 4 experiments (1, 10, 11).

**Immunohistochemistry (IHC)**. The CHTN BrCaProg1 Microarray slides were obtained from CHTN (Cooperative Human Tissue Network); the slides were firstly fixed by 3.7% formaldehyde at 37ºC for 15 min, then permeabilized by 1% BSA+0.2% Triton X-100 in PBS for 1 hr, blotted with 40μg/ml (dilue 1:20 or 1:50) of rabbit antibody for either PGC1β or HKDC1 for 2 hours, and washed three times.FITC labeled anti-rabbit secondary antibody (1:100-1:200) was added for blotting for another 1 hour, and after thorough washing, the slides were visualized and photographed; more than 60 cells in each group were quantitated for protein expression by using Image J. software (1).

**Immunostaining**. The cells were transferred to cover slips coated with 0.1% gelatin, then stained by MitoTracker Red (#PA-3017 from Lonza, in option). The cells were then fixed by 3.7% formaldehyde at 37ºC for 15 min, permeabilized by 1% BSA+0.2% Triton X-100 in PBS for 1 hour, and then blotted with 40μg/ml (dilute 1:50) of mouse monoclonal antibody for either Ki-67 (MIB-1) or HKDC1 for 2 hours. The cells were then washed three times and the FITC labeled anti-mouse (or anti-rabbit) secondary antibody (1:100) was added for blotting for another 1 hour. After thorough washing, the slides were visualized and photographed, and the nuclei of cells were stained with 4’,6-diamidino-2-phenylindole dihydrochloride (DAPI, #D9542, from Sigma), and the positive Ki-67 cells were quantitated (1, 6).

**Animals**. Balb/c athymic nude male mice (6 weeks old) were obtained from the Guangdong Medical Animal Center. All procedures involving mice were conducted in accordance with NIH regulations concerning the use and care of experimental animals and were approved by the Institutional Animal Care and Use Committee (from Peking University Shenzhen Hospital). The 2x10^6^ viable treated tumor cells were washed, harvested in PBS, and then injected into the lateral tail vein in a volume of 0.1ml. Mice were monitored for changes in body weight and sacrificed when values fell below 20% of their initial weight. The lungs of sacrificed mice were isolated and fixed in 10% formalin. The number of surface metastases per lung was determined under a dissecting microscope. Formalin-fixed, paraffin-embedded tumor tissue from the lungs were sectioned to 4mm thickness, and the histopathological analyses were performed with H&E staining. Images were taken using a Carl Zeiss MIRAX MIDI slide scanner, and analyses were performed using a 3DHISTECH Pannoramic Viewer. The tumor tissues were isolated for *in vivo* monitoring of superoxide anion release, and gene expression in tumor tissues was measured by real time PCR for mRNA and Western Blotting for protein levels (1, 3, 6).

**In vivo superoxide release**. Superoxide anion (O_2_^.-^) release from tumor tissues was determined using a luminol-EDTA-Fe enhanced chemiluminescence (CL) system supplemented with DMSO-TBAC (Dimethyl sulfoxide-tetrabutyl-ammonium chloride) solution for extraction of released O_2_^.-^ from tissues, as described previously (7). Superoxide levels were calculated from the standard curve generated by the xanthine/xanthine oxidase reaction (3).

**Statistical analysis.** The data was given as mean ± SEM; all of the experiments were performed at least in quadruplicate unless otherwise indicated. The one-way ANOVA followed by the Bonferroni post hoc test was used to determine statistical significance of different groups. The mouse survival curve was determined by Kaplan-Meier survival analysis using SPSS 22 software and a *P* value < 0.05 was considered significant (3).

REFERENCES

1. Zhang H, Li L, Chen Q, Li M, Feng J, Sun Y, Zhao R, Zhu Y, Lv Y, Zhu Z, et al. PGC1beta regulates multiple myeloma tumor growth through LDHA-mediated glycolytic metabolism. *Mol Oncol.* 2018;12(9):1579-95.

2. Zou Y, Lu Q, Zheng D, Chu Z, Liu Z, Chen H, Ruan Q, Ge X, Zhang Z, Wang X, et al. Prenatal levonorgestrel exposure induces autism-like behavior in offspring through ERbeta suppression in the amygdala. *Mol Autism.* 2017;8(46.

3. Zhang H, Li L, Li M, Huang X, Xie W, Xiang W, and Yao P. Combination of betulinic acid and chidamide inhibits acute myeloid leukemia by suppression of the HIF1alpha pathway and generation of reactive oxygen species. *Oncotarget.* 2017;8(55):94743-58.

4. Metivier R, Penot G, Hubner MR, Reid G, Brand H, Kos M, and Gannon F. Estrogen receptor-alpha directs ordered, cyclical, and combinatorial recruitment of cofactors on a natural target promoter. *Cell.* 2003;115(6):751-63.

5. Ceradini DJ, Yao D, Grogan RH, Callaghan MJ, Edelstein D, Brownlee M, and Gurtner GC. Decreasing intracellular superoxide corrects defective ischemia-induced new vessel formation in diabetic mice. *J Biol Chem.* 2008;283(16):10930-8.

6. Zhang H, Lu J, Jiao Y, Chen Q, Li M, Wang Z, Yu Z, Huang X, Yao A, Gao Q, et al. Aspirin Inhibits Natural Killer/T-Cell Lymphoma by Modulation of VEGF Expression and Mitochondrial Function. *Front Oncol.* 2018;8(679.

7. Yao D, Shi W, Gou Y, Zhou X, Yee Aw T, Zhou Y, and Liu Z. Fatty acid-mediated intracellular iron translocation: a synergistic mechanism of oxidative injury. *Free Radic Biol Med.* 2005;39(10):1385-98.

8. Kong D, Zhan Y, Liu Z, Ding T, Li M, Yu H, Zhang L, Li H, Luo A, Zhang D, et al. SIRT1-mediated ERbeta suppression in the endothelium contributes to vascular aging. *Aging Cell.* 2016.

9. Somasundaram K, and El-Deiry WS. Inhibition of p53-mediated transactivation and cell cycle arrest by E1A through its p300/CBP-interacting region. *Oncogene.* 1997;14(9):1047-57.

10. Han HJ, Russo J, Kohwi Y, and Kohwi-Shigematsu T. SATB1 reprogrammes gene expression to promote breast tumour growth and metastasis. *Nature.* 2008;452(7184):187-93.

11. Yu OM, Benitez JA, Plouffe SW, Ryback D, Klein A, Smith J, Greenbaum J, Delatte B, Rao A, Guan KL, et al. YAP and MRTF-A, transcriptional co-activators of RhoA-mediated gene expression, are critical for glioblastoma tumorigenicity. *Oncogene.* 2018.

**Table S1. Sequences of primers for the real time quantitative PCR (qPCR)**

| Gene | Species | Analysis | Forward primer (5'→3') | Reverse primer (5'→3') |
| --- | --- | --- | --- | --- |
| β-actin | Human | mRNA | gatgcagaaggagatcactgc | atactcctgcttgctgatcca |
| PGC1α | Human | mRNA | catgcaaatcacaatcacagg | ggtcatcgtttgtggtcagat |
| PGC1β | Human | mRNA | tgctagcctcaccaaacactt | ttcttcctcttcctcctctgg |
| PPRC1 | Human | mRNA | ggggttgtcattgaactcaga | tcttcttcttcctgccctttc |
| HK1 | Human | mRNA | cctgcatctctgacttcttgg | acgcagtctgttgccttaaaa |
| HK2 | Human | mRNA | gatttcaccaagcgtggacta | aagccctaagtgttgcaggat |
| HK3 | Human | mRNA | agggtttcaaggcatcagact | tcctcatagccacaggacatc |
| HK4 | Human | mRNA | tatcaaacggagaggggactt | cattctgcatctcctccatgt |
| HKDC1 | Human | mRNA | acgagtttgacctggacattg | ccatctcgatgttcctcatgt |
| SREBP1 | Human | mRNA | actgaggtggaggacacactg | cttgctgtcctcaaagactgg |
| SREBP2 | Human | mRNA | agtctggtggacaatgaggtg | tcatccaatagagggcttcct |
| HKDC1 | Human | ChIP | gagatgggaggatcacctgag | ggagcatttaccaatcccatt |

FIGURE S1.

**Figure S1. Gene expression in normal, breast tumor and metastatic cells.** Different cells, including primary HMECs, MCF7 and MDA231 cells were infected by either empty (CTL) or shPGC1β lentivirus, and the cells were harvested for gene expression analysis. (a) The mRNA levels for PGC1α, PPRC1, SREBP1 and SREBP2, n=4. (b) The mRNA levels for HK1, HK2, HK3 and HK4, n=4. *, *P*<0.05, vs HMECs group; ¶, *P*<0.05, vs MCF7/CTL group. Results are expressed as mean ± SEM.

FIGURE S2

**Figure S2.** **HKDC1 overexpression potentiates breast tumor growth in *in vivo* xenograft tumor development, while HKDC1 knockdown reverses this effect.** The nude mice were injected with treated MDA231 cells through the tail vein for *in vivo* xenograft tumor development study, and the treated mice were sacrificed for further analysis. (c) The tumor tissues from the lung were isolated for mRNA analysis by qPCR, n=4. *, *P*<0.05, vs CTL group. (d) Superoxide anion release from tumor tissues, n=5, *, *P*<0.05, vs CTL group. (c,d) Mice were killed upon 20% weight loss, and the lungs were harvested for terminal analysis. The metastatic tumor nodules from the lungs were counted, and then the formalin-fixed, paraffin-embedded tumor tissue from the lung was sectioned to 4mm thickness. Histopathological analyses were performed with H&E staining. Images were taken using a Carl Zeiss MIRAX MIDI slide scanner, and the lung tumor spots were analyzed using a 3DHISTECH Pannoramic Viewer. (c) Tumor colony formation in lung, n=7. *, *P*<0.05, vs CTL group; ¶, *P*<0.05, vs ↑PGC1β group. (d) Quantitated lung tumor spots, n=5. *, *P*<0.05, vs CTL group; #, *P*<0.05, vs shPGC1β group. Results are expressed as mean ± SEM.
